# Pyrethroid insecticides and their environmental degradates in repeated duplicate-diet solid food samples of 50 adults

**DOI:** 10.1038/jes.2016.69

**Published:** 2016-12-14

**Authors:** Marsha K. Morgan, Denise K. MacMillan, Dan Zehr, Jon R. Sobus

**Affiliations:** 1 National Exposure Research Laboratory, US EPA, Research Triangle Park, North Carolina, USA; 2 National Health and Environmental Effects Research Laboratory, US EPA, Research Triangle Park, North Carolina, USA.

**Keywords:** adults, degradates, dietary, food, pyrethroids, temporal

## Abstract

Previous research has reported concurrent levels of pyrethroid insecticides and their environmental degradates in foods. These data raise concerns about using these same pyrethroid degradates found in the diet as urinary biomarkers of exposures in humans. The primary objective was to quantify levels of selected pyrethroids and their environmental degradates in duplicate-diet solid food samples of 50 adults over a six-week monitoring period. The study was conducted at the US EPA’s Human Studies Facility in North Carolina and at participants’ residences in 2009–2011. Participants collected duplicate-diet solid food samples on days 1 and 2 during weeks 1, 2, and 6 of the monitoring period. These samples were collected over three consecutive time periods each sampling day. A total of 782 food samples were homogenized and analyzed by LC/MS/MS for seven pyrethroids (bifenthrin, *λ*-cyhalothrin, cyfluthrin, cypermethrin, *cis*-deltamethrin, esfenvalerate, and *cis/trans*-permethrin) and six pyrethroid degradates. Results showed that 49% and 2% of all the samples contained at least one target pyrethroid or pyrethroid degradate, respectively. *Cis/trans*-permethrin (20%) and bifenthrin (20%) were the most frequently detected pyrethroids. The results suggest that the pyrethroid degradates were likely not present in sufficient levels in the diet to substantially impact the adults’ urinary biomarker concentrations.

## INTRODUCTION

Pyrethroids are a class of insecticides that are used for crop protection worldwide.^1,2^ These lipophilic insecticides account for more than 17% of the global agrochemical market.^1^ In the United States (US), at least 18 different pyrethroid insecticides are currently registered by the US Environmental Protection Agency (EPA) for use on domestic crops for human consumption.^3^ However, no published data are currently available on the total amount of pyrethroid insecticides that are applied on US croplands, including residential gardens, each year.^3^

Research has shown that humans can be exposed to pyrethroids through the inhalation, ingestion, and dermal routes.^2,4,5^ However, dietary ingestion is likely the major exposure route to these insecticides in the general US adult population.^4–5^ This is supported by several studies reporting measureable residues of a number of different current-use pyrethroids in duplicate-diet samples or in individual food items (e.g., lettuce, blueberries, butter, and sour cream).^6–14^ In these studies, pyrethroid insecticides were generally detected at much higher levels in solid foods compared to liquid foods (e.g. beverages).^6,10,13,14^ Current-use pyrethroids detected in these solid food samples included bifenthrin, cyfluthrin, *λ*-cyhalothrin, cypermethrin, *cis*-deltamethrin, esfenvalerate, and *cis/trans*-permethrin.

Few published data are available on the concurrent levels of these pyrethroid insecticides and their environmental degradates in foods.^7,11^ In a recent study by Chen et al.,^11^ they reported measureable levels of cypermethrin, *λ*-cyhalothrin, *cis*-deltamethrin, esfenvalerate, and/or permethrin, and their common degradation product, 3-phenoxybenzoic acid (3-PBA), in eight different produce samples, collected in California between 2010 and 2011. This information raises concerns about using 3-PBA and perhaps other pyrethroid degradates found in the diet as urinary biomarkers of exposure in humans.

After oral ingestion, pyrethroids are metabolized quickly and are mainly renally eliminated as polar metabolites (e.g., 3-PBA) in humans with an urinary excretion half-life of less than 10h.^15–17^ For the pyrethroid degradates, no published (oral) toxicokinetic data exist based on human exposure studies. Previous biomonitoring studies have found several different pyrethroid metabolites (i.e., 3-PBA and *cis/trans*-3-(2,2-dichlorovinyl)-2,2-dimethylcyclo-propane carboxylic acid (*cis/trans*-DCCA)) in the urine of the general US adult population.^5,18–21^ It is currently unknown whether pyrethroid degradates in the diet are substantially contributing to pyrethroid metabolite levels in the urine of adults in the general US population.

The Pilot Study to Estimate Human Exposures to Pyrethroids using an Exposure Reconstruction Approach (Ex-R study) investigated the temporal exposures (over 6 weeks) of 50 North Carolina (NC) adults to selected pyrethroid insecticides and their environmental degradates in several different media (e.g., food, drinking water, dust, and floor wipes) at homes in 2009–2011.^21^ In this current work, we quantified the levels of seven current-use pyrethroids and six of their environmental degradates (Table 1) in 782 duplicate-diet solid food samples of 50 Ex-R adults over a six- week monitoring period. We also estimated the maximum daily dietary intake doses of the adults to each target pyrethroid.

## MATERIALS AND METHODS

### Study Cohort

A detailed description of the Ex-R study design and sampling methodology can be found in Morgan et al.^21^ Briefly, this study was performed at the US EPA’s Human Subjects Facility (HSF) located in Chapel Hill, NC and at the participants’ residences within a 40 mile radius of the HSF.^21^ Recruitment of adult participants began in October 2009 and ended in March 2011. Field sampling occurred at the participants’ residences from November 2009 to May 2011. The adults, ages 19–50 years old, collected their own duplicate-diet solid food samples and completed daily food diaries over a 6-week monitoring period. All protocols and procedures for the Ex-R study were approved by the University of North Carolina’s Institutional Review Board (study number: 09–0741).^21^ In addition, the adults signed informed consent forms before participating.

### Collection of Duplicate-Diet Solid Food Samples

The duplicate-diet solid food samples consisted of duplicate amounts of all the consumed solid foods (excluding beverages) that the participants’ ate on day 1 (Sunday) and day 2 (Monday) during weeks 1, 2, and 6 of the six-week monitoring period.^21^ These samples were collected by each participant over three, consecutive time periods (period 1 = 4:00 am −11:00 am; period 2 = 11:00 am - 5:00 pm; and period 3 = 5:00 pm - 4:00 am) each sampling day. Up to 18 individual food samples (6 per sampling week) were collected by each participant over the monitoring period. Solid foods were defined as any edible items that were solid, semi-solid, or contained solids (e.g., hamburger, salad, vegetable soup, smoothie, and ice cream). Inedible parts of foods such as chicken bones, banana peels, and apple cores as well as medicines/vitamins were excluded from these samples. The participants also completed daily food diaries that listed the specific food items that they consumed during each sampling time period.

For each food sample, the participants placed duplicate amounts of their consumed solid food items into a re-sealable polyethylene bag (31 × 31 cm; Uline Shipping Supply Specialists, Pleasant Prairie, WI) and then into a larger re-sealable polyethylene bag (41 × 41 cm). The collection date of each food sample was written with a pen on the provided label on the outside polyethylene bag. The food samples were stored in portable thermoelectric coolers (Princess International, Brooklyn, NY, USA or Vinotemp, Irvine, CA, USA) until the participants returned the coolers to the HSF on day 3 (Tuesday) between 8:00 am–11:00 am of each sampling week.^21^ At the HSF, US EPA researchers weighed each food sample. These researchers then transported the food samples in study coolers with blue ice to an US EPA laboratory in Research Triangle Park (RTP), NC, USA.

### Food Sample Preparation, Extraction, and Analysis

Food samples were homogenized (for up to 1 min) using a vertical cutter mixer (Robot Coupe R4N-D or R10-Ultra) or a high-speed blender (Waring MBB518 Professional Food/Beverage). The blender was only used for a few types of food items (i.e., soups and purees). Samples containing a single food item with a smooth texture (i.e., peanut butter and ice cream) were not homogenized. Up to six replicates (12 g each) of each food sample were then transferred into separate, pre-cleaned amber glass jars (30 ml) with lids. All replicates were stored in US EPA laboratory freezers (⩽ −20 °C) until analysis.

The homogenized food samples were extracted using the QuEChERS (quick, easy, cheap, effective, rugged, and safe) technique that was modified for use in complex food mixtures.^22,23^ For each study sample, a 12 g replicate was thawed, and a 2 g aliquot was removed and transferred to a vial. The 2 g aliquot was then spiked with internal standards (^13^C_6_-cypermethrin, ^13^C_6_-*λ*-cyhalothrin, ^13^C_6_-*cis*-permethrin, ^13^C_6_-*trans-*permethrin, ^13^C_6_-3-PBA, ^13^C_6_-4-fluoro-3-phenoxybenzoic acid (^13^C_6_-4-F-3-PBA), and ^13^C_6_-d_1_-*cis*-DCCA [Cambridge Isotope Laboratories, Andover, MA, USA]) and 2 ml of acetonitrile was added. The sample was mechanically shaken for 1–2 min. to completely wet the contents, and then MgSO_4_ (800 mg) and NaCl (200 mg) were added. The sample was vortexed to fully disperse the solids in the mixture, and then centrifuged at 4000 rpm (3220 × g) for 5 min. Next, an aliquot of the supernatant (1 ml) was added to a clean-up tube containing 150 mg MgSO_4_/carbon black (Supelclean ENVI-carb 120/400 (Supelco, Sigma-Aldrich, Bellefonte, PA, USA, 30:1; w/w) and 300 mg bulk C-18 (Discovery DSC-C18, Supelco, Sigma-Aldrich). The clean-up tube was vortexed for 30 s. and then centrifuged at 15,000 rpm (26,000 *g*) for 10 min. An aliquot (300 μl) of the resulting extract was diluted 1:1 with 0.1% formic acid, filtered through a PTFE filter (0.2 μm), and stored at ~ 4 °C until analysis.

The extracts were analyzed to quantify levels of the target pyrethroids and their environmental degradates (Table 1) using electrospray ionization liquid chromatography/tandem mass spectrometry (LC/MS/MS) on a 4000 QTrap linear ion trap mass spectrometer (AB Sciex, Framingham, MA, USA) in positive (pyrethroids) or negative (degradates) ion modes. All analytical standards were purchased from Chem Services (West Chester, PA, USA) with the exception of 4-F-3-PBA (Cambridge Isotope Laboratories, Andover, MA, USA) and 2-methyl-3-phenylbenzoic acid (MPA) (FMC Agricultural Products Group, Philadelphia, PA, USA). Instrument parameters are given in Supplementary Table 1. Separation was performed using a Hypersil Gold 50 × 2.1 mm column (1.9 μ) (Thermo Fisher Scientific, Waltham, MA, USA) with gradient elution (Supplementary Table 2). The mobile phase used for the parent pyrethroids consisted of (A) 25:75 5 mM ammonium acetate:methanol with 0.1% formic acid, and (B) 5 mM ammonium acetate in methanol with 0.1% formic acid. For the pyrethroid degradates, the mobile phases were the same as mentioned above, except formic acid was not included. Multiple reaction monitoring with two characteristic transitions per target analyte was used for definitive identification (Supplementary Table 3). Quantitation was obtained by internal calibration using isotope dilution and matrix-matched curves made by spiking the control matrix with internal standards and target analytes prior to extraction. The limits of quantitation (LOQs) for the target analytes were set at the concentration of the lowest calibration standard. Estimated limits of detection (LODs) were set at concentrations that gave a signal-to-noise ratio of at least 3 and were ~ 2–5 times below LOQs. The LODs and LOQs for the target analytes are provided in Table 2. Calibration ranges were from the LOQ to 200 ng/g for all analytes. Curves were constructed using quadratic equations with 1/x weighting. Additional details of the method validation are included in the Supplementary Information section.

### Quality Assurance and Quality Control Procedures

Field and laboratory quality control samples were used to determine the overall quality of food sample collection, processing, extraction, and analysis. A control food mixture was prepared in-house to be used as the medium for the field/laboratory blank and spike samples. Approximately 5 kg of the control food mixture (5% fat) was made using a slightly modified method by Rosenblum et al.^24^ The food mixture contained applesauce, bread, cake, cereal, cheese, fruit cocktail, lunchmeat, margarine, mayonnaise, pizza, potato chips, and vegetables that were purchased from local grocery stores. Organic food items were used (when available) to reduce background contamination of pyrethroid insecticides. The control food mixture was homogenized in 1 kg batches using a vertical cutter mixer (Robot Coupe R10-Ultra). Replicates of control food (12 g each) were placed into pre-cleaned amber glass jars (30 ml) and stored in ⩽ − 20 °C in freezers.

The field QC samples consisted of blanks and three spikes (low, medium, and high levels) that were assigned to 10% of the study participants during days 1–2 of each sampling week. A blank or spike sample contained 12 g of the control food mixture (above) that was spiked with internal standards by using 100 μl of a mixed standard (500 ng/ml in methanol) for a final concentration of 4.2 ng/g. For the field spike samples only, they were also spiked with 20, 100, or 200 μl of a solution (in methanol) of the target pyrethroids for a final concentration of 0.9, 4.5, or 8.9 ng/g and the pyrethroid degradates for a final concentration of 4.5, 22.5, or 45 ng/g, respectively. The concentration of the mixed standard used for spiking the field samples was 500 ng/ml for the pyrethroids and 2500 ng/ml for the degradates. Each field blank or field spike was stirred thoroughly with a spatula prior to being transferred into a re-sealable polyethylene bag (8 × 13 cm) and then into another polyethylene bag (8 × 13 cm). These field QC samples were placed into a portable thermoelectric cooler (plugged into an electric wall outlet) in a study room at the HSF during each sampling period (Sunday–Monday). The samples were stored in EPA laboratory freezers (⩽ −20 °C) until analysis. The field blanks (*n* = 18) were below the LODs for all pyrethroids and their environmental degradates in food, except for *cis*-permethrin and *trans*-permethrin that co-occurred in two samples. For the medium and high field spike samples, mean percent recoveries of the target analytes were within empirically determined percent recovery acceptance limits, except for *λ*-cyhalothrin, *cis*-permethrin, *trans*-permethrin, 3-PBA, and *cis*-DBCA (Supplementary Table 4). For the low field spike samples, the recovery results were inconsistent and highly variable for all analytes (data not shown) most likely due to the low spiked volume (20 μl) which caused incomplete mixing of the samples. A detailed discussion of the field spike results can be found in the Supplementary Information section.

The laboratory (batch) QC samples consisted of laboratory blanks, sample duplicates (duplicate extraction of a study sample), analytical duplicates (duplicate analysis of a study sample extract), laboratory control samples (LCS; spiked control mixture) and matrix spikes (MSS; spiked study samples). The LCS and MSS were spiked at 10 ng/g. The laboratory blanks (*n* = 50) were all below the LODs for the target analytes in food, except for *cis*-permethrin in one sample and *trans*-permethrin in three samples. Mean values for the laboratory blanks were below the LOD for each isomer, so no background correction was made. Mean relative percent differences between sample duplicates or analytical duplicates for the parent pyrethroids were less than 7% in the food samples. For the pyrethroid degradates, mean relative percent differences between the sample duplicates or analytical duplicates were less than 1%. Mean percent recoveries for the LCS and MSS were within the empirically determined percent recovery acceptance limits for all target analytes (Supplementary Table 4). In addition, the mean percent recoveries of the target analytes for the MSS were within 5% of the mean percent recoveries for the LCS, except for bifenthrin. For this analyte, the mean percent recovery for the MSS was 15% lower than the mean percent recovery for the LCS.

### Statistical Analyses

For each target analyte, all data values below the LOD in the food samples were replaced by the LOD/$\sqrt{2}$ as described in Verbovsek.^25^ Descriptive statistics were calculated for the analytes in the food samples using JMP version 12.1 (SAS Institute, Cary, NC, USA).

The estimated maximum daily dietary intake doses (ng/kg/day) of the Ex-R adults to each target pyrethroid insecticide (*D*) was calculated using the following equation:

$$D=\frac{\sum_{t=1}^{3}\left(F_{t}\times M_{t}\right)}{B}$$

In this equation, Ft (concentration (ng/g) of each target pyrethroid in a food sample) was multiplied by *M*_*t*_ (mass of the same food sample (g)). The subscript t denotes whether the sample was the first, second, or third sample on a given sampling day. Then, the amount of each target pyrethroid in the participant’s food samples (up to three) was summed over a sampling day (ng/d) and divided by *B* (body weight (kg)).

## RESULTS

Table 3 provides the summary statistics for the target pyrethroids and their environmental degradates measured in the duplicate-diet solid food samples of 50 Ex-R participants over the six-week monitoring period. For the pyrethroids, *trans*-permethrin (21%), bifenthrin (20%), *cis*-permethrin (19%), and *cis*-deltamethrin (17%) were detected the most often in the food samples. At the 95th percentile, the levels of *cis*-permethrin (5.2 ng/g) and *trans-*permethrin (5.4 ng/g) were at least two times greater compared to the levels of the other pyrethroids (⩽1.9 ng/g) in the samples. The highest maximum concentration of the pyrethroids occurred for esfenvalerate (358 ng/g) in one participant’s food sample. In comparison to the parent pyrethroids, the degradates were not frequently detected (<3%) in the food samples (Table 3). MPA (bifenthrin degradate) was not detected in any of these samples. The highest maximum levels of the degradates occurred for *trans*-DCCA (38.3 ng/g) and *cis*-DCCA (8.3 ng/g) in the same food sample of one participant.

**Figure 1.**
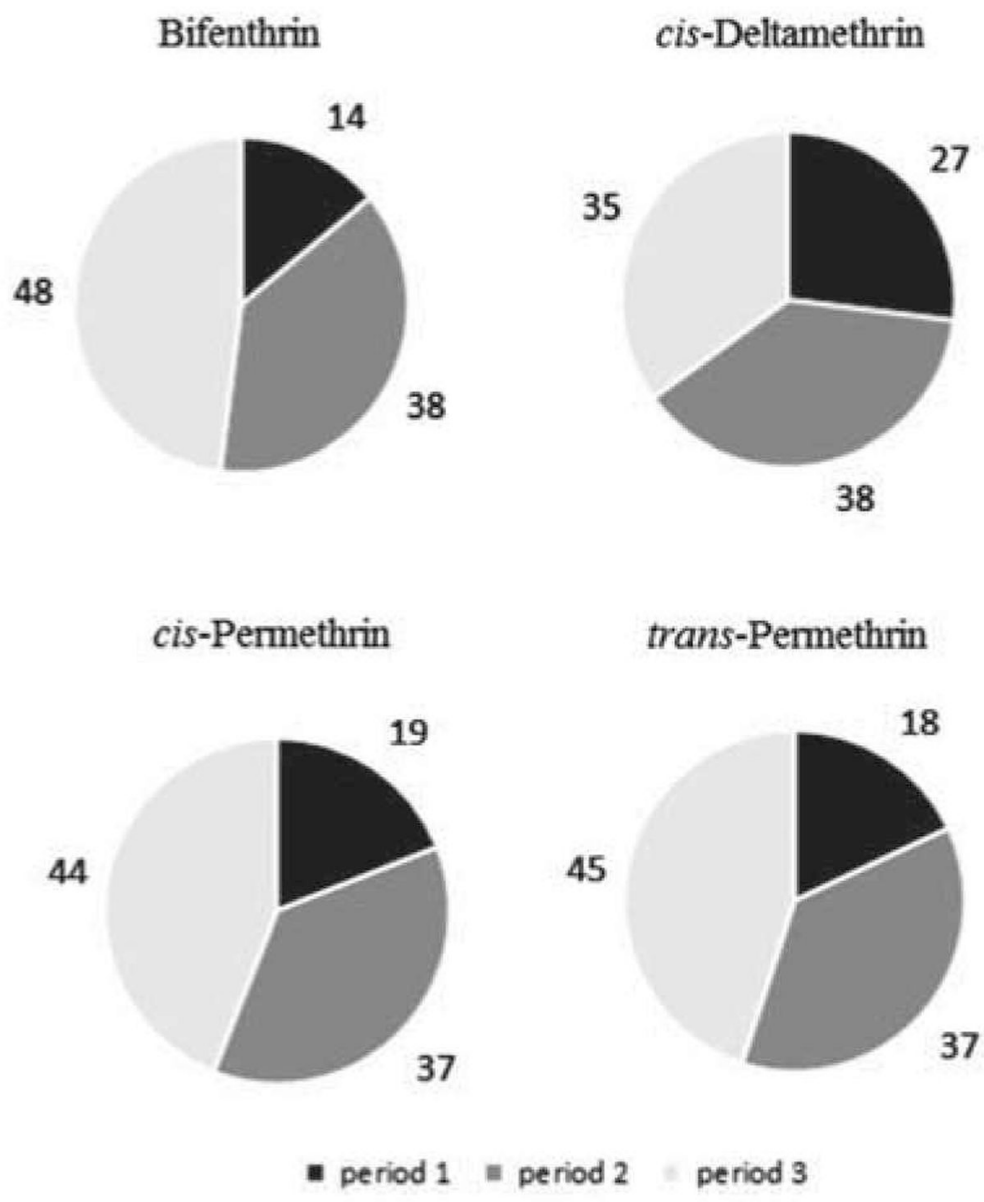
Percentage of duplicate-diet solid food samples with detectable levels of bifenthrin, *cis*-deltamethrin, *cis*-permethrin, and *trans*-permethrin by sampling time period.^a,b,c a^Includes the four most frequently detected (⩾17%) pyrethroids measured in the duplicate-diet solid food samples. ^b^Data are shown for food samples that have detectable levels (⩾ LOD) of each pyrethroid by time period (period 1 = 0400–1100 hours, period 2 = 1100–1700 hours, and period 3 = 1700–0400 hours). ^c^Number of food samples: bifenthrin (*n* = 156), *cis*-deltamethrin (*n* = 129), *cis*-permethrin (*n* = 145), and *trans*-permethrin (*n* = 164).

Table 4 presents the summary statistics for the levels (⩾ LOD) of the four most frequently detected pyrethroids (bifenthrin, *cis*-deltamethrin, *cis*-permethrin, and *trans*-permethrin) in the food samples. Mean levels of *cis*-permethrin (9.1 ±20.0 ng/g) and *tran*-spermethrin (8.9 ±22.2 ng/g) were at least four time greater compared to mean levels of bifenthrin (1.8 ±2.8 ng/g) and *cis*-deltamethrin (1.9± 2.4 ng/g) in the food samples. At the 95th percentile, the levels of *cis*-permethrin (52.5 ng/g) and *trans*-permethrin (48.6 ng/g) were at least five times higher than the levels of the other two pyrethroids (⩽8.2 ng/g). Figure 1 presents the levels of these four pyrethroids in the participant’s food samples by sampling time period (1, 2, or 3). The results show that these four pyrethroids were consistently detected the least often in the food samples during period 1 (4:00 am - 11:00 am) compared with period 2 (11:00 am - 5:00 pm) and period 3 (5:00 pm - 4.00 am). These pyrethroids were found the most often in the participants’ food samples during period 3, except for *cis*-deltamethrin (period 2).

**Figure 2.**
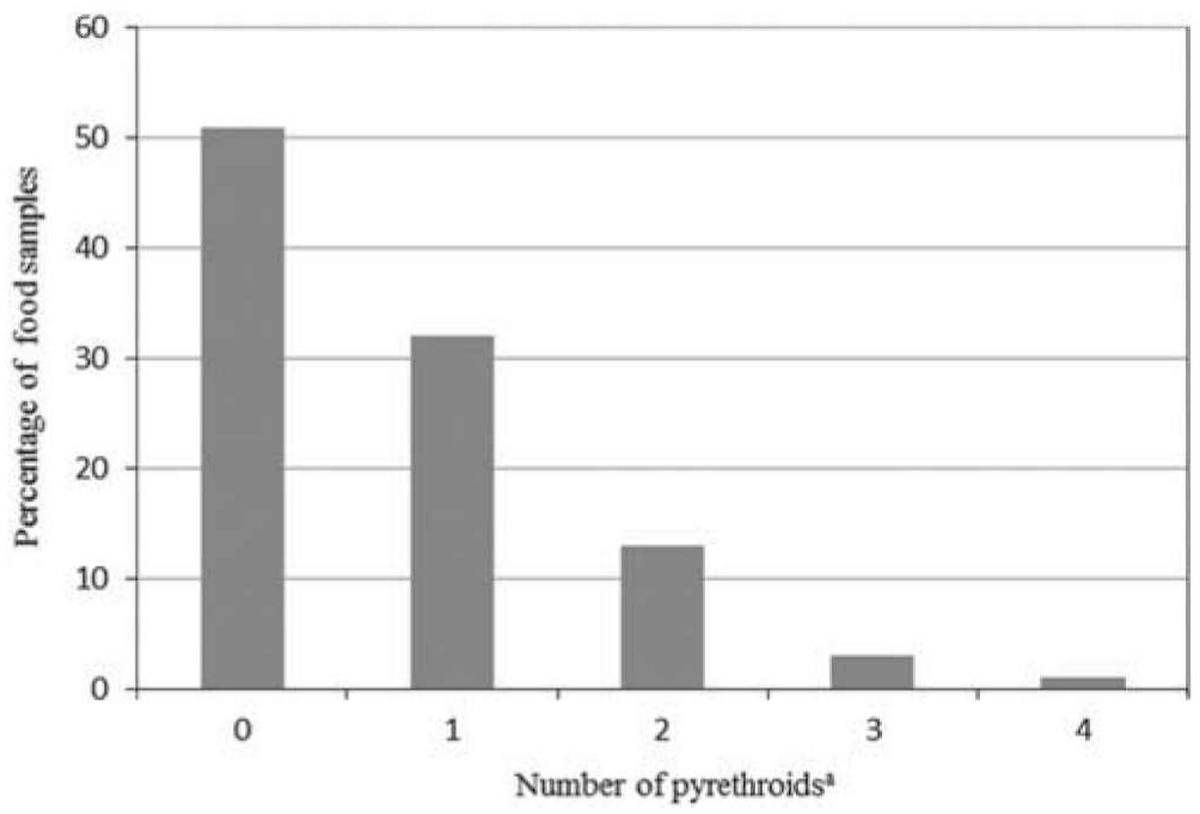
Co-occurrence of the target pyrethroid insecticides in the duplicate-diet solid food samples of Ex-R adults. ^a^*cis*-and *trans*-permethrin were counted as one pyrethroid insecticide.

The co-occurrence of the target pyrethroids in the food samples of the Ex-R adults over the six-week monitoring period is presented in Figure 2. In this figure, the results show that 49% of all the food samples contained at least one or more target pyrethroids. In particular, 32% of the participants’ food samples contained one target pyrethroid. In addition, 13% of the adult food samples contained at least two different target pyrethroids. Only 3% and 1% of the food samples had three or four different target pyrethroids, respectively.

Table 5 presents the estimated adults’ maximum dietary exposures (ng/day) and maximum dietary intake doses (ng/kg/day) to the target pyrethroid insecticides and compares these estimates to available oral reference doses (RfDs) in the US EPA’s Integrated Risk Information System (IRIS).^26^ In IRIS, oral RfDs currently exist only for bifenthrin, *λ*-cyhalothrin, cypermethrin, and permethrin. The participants’ maximum dietary intake doses were the highest for the combined isomers of *cis/trans*-permethrin (2,115 ng/kg/day), followed by esfenvalerate (1,784 ng/kg/day). The estimated adults’ maximum dietary intake doses to bifenthrin, *λ*-cyhalothrin, cypermethrin, and permethrin were all well below the established oral RfDs in IRIS.

## DISCUSSION

Previous research has suggested that the diet is likely the major source of exposure to pyrethroids insecticides in the general adult population worldwide.^4,5,27^ Few data exist on the temporal levels of current-use pyrethroid insecticides in the everyday diets of adults.^10,12^ Our study results showed that the individual target pyrethroids were not detected often (1–21%) in the duplicate-diet solid food samples of 50 Ex-R adults over the 6-week monitoring period. However on a cumulative basis, 49% of the participants’ food samples contained at least one of the target pyrethroids. Based on these data, 100% of the Ex-R participants were intermittently exposed to several different pyrethroids in their normal diets during the six-week monitoring period. Our study results are similar to Melnyk et al.^12,28^ that also showed low detection frequencies (LOD range = 0.05–0.8 ng/g) for bifenthrin (30%), cyfluthrin (4%), cypermethrin (11%), *cis*-deltamethrin (1%), esfenvalerate (10%), and *cis/trans*-permethrin (33%) in 67 solid food samples composited by eating event (breakfast, lunch, dinner, and snack) over several days for nine Hispanic women in Florida USA in 2008. In an earlier study conducted by Riederer et al.,^10^ the authors reported higher detection frequencies (LOD range = 0.4–0.8 ng/g) than our study for cyfluthrin (59%), cypermethrin (67%), *cis*-deltamethrin (28%), and *cis/trans*-permethrin (43%) in duplicate diet samples (composited by food group) of 12 adults collected over eight sampling days in Georgia USA in 2005–2006. Interestingly in that study, the authors reported that measureable levels of these pyrethroid residues were found in several different types of foods (i.e., fruits, vegetables, legumes, grains, meats, and dairy).^10^ This information emphasize the importance of collecting total diet samples (i.e., not just produce) from adults to assess their daily dietary exposures to pyrethroids. As data are limited, more research is necessary to determine the cumulative and temporal dietary exposures of adults to current-use pyrethroid insecticides.

Another important study finding was that the detectable levels of the target pyrethroids substantially varied by sampling time frame (period 1, 2, or 3) over a day. The most frequently detected pyrethroids (bifenthrin, *cis*-deltamethrin, *cis*-permethrin, and *trans*-permethrin) were found less often in food consumed during period 1 compared to periods 2 and 3. In support of our study results, Melnyk et al.^12,28^ also reported that the mean intake levels of several different pyrethroids were substantially different by type of adult meal sampled (breakfast, lunch, or dinner). In that study, the authors found that the mean intake levels of permethrin, cypermethrin, and esfenvalerate were the lowest in the participants breakfast meals compared to their lunch/dinner meals. This information suggests that people’s eating patterns are likely an important factor influencing their dietary exposures to pyrethroid insecticides over a day.

Few data are available on the estimated dietary intake doses to single or multiple pyrethroid insecticides derived from the actual consumed diets of adults, globally.^10^ Assuming 100% absorption, our estimated adults’ maximum dietary intake doses to bifenthrin (63.1 ng/kg/day), *λ*-cyhalothrin (69.7 ng/kg/day), cypermethrin (393 ng/kg/day), and permethrin (2,115 ng/kg/day) were all well below the corresponding oral RfDs listed in IRIS (Table 5).^26^ Since oral RfDs are not currently available for cyfluthrin, *cis*-deltamethrin, or esfenvalerate in IRIS,^26^ we could not determine if the estimated adults’ dietary intake doses to these insecticides (Table 5) were below a level of concern. Only one other smaller duplicate-diet study was found by Reiderer et al.^10^ that reported estimated dietary intake doses to cypermethrin (1400 ng/kg/day), *cis*-deltamethrin (2700 ng/kg/day), and permethrin (1500 ng/kg/day) for 12 adults in Georgia USA in 2005–2006.

Few published studies have measured the concurrent levels of pyrethroids and their environmental degradates (commonly used as urinary biomarkers) in individual food items (i.e., produce)^7,11^ and none in duplicate-diet samples. Our study results showed that 3-PBA, 4-F-3-PBA, *cis*-DBCA, *cis*-DCCA, *trans*-DCCA, and MPA were not frequently detected (<3%) in the duplicate-diet solid food samples of 50 Ex-R participants over the six-week monitoring period. This information suggests that these pyrethroid degradates were probably not present in sufficient levels in the diet to substantially influence the Ex-R adults’ urinary pyrethroid biomarker concentrations. Our results are in agreement with Li et al.^7^ that recently reported measureable levels of several different pyrethroids in samples of apples, grapes, and lettuce purchase from local grocery stores in Durham NC, but only the pyrethroid degradates, *cis*-DCCA and *trans*-DCCA, occurring in one lettuce sample.

Our study had some limitations. Because of participant burden and budget constraints, the Ex-R study only collected duplicate-diet solid food samples (which excluded beverages). Therefore, it is possible that we may have underestimated the total daily dietary exposures and intake doses of the Ex-R adults to these individual pyrethroids. However, previous research has indicated that beverages were a minor contributor to the dietary exposure of adults to pyrethroid insecticides.^6,9,29^ Another study limitation was that the Ex-R participants’ solid food samples were collected over three, consecutive time frames each sampling day, so we were unable to determine the specific food items that had detectable levels of the pyrethroid residues.

## CONCLUSIONS

On basis of the food sample data, our study results showed that the Ex-R adults were likely intermittently exposed to several different pyrethroid insecticides in their diets during the six-week monitoring period in NC in 2009–2011. In addition, the results suggests that the measured pyrethroid degradates (3-PBA, 4-F-3-PBA, *cis*-DBCA, *cis*-DCCA, and trans-DCCA) were likely not present in sufficient levels in the diet to substantially impact the adults’ urinary biomarker concentrations.^30^

## Supplementary Material

s1

## Figures and Tables

**Table 1.** Target pyrethroid insecticides and their environmental degradates measured in the duplicate-diet solid food samples.

*Pyrethroid*	*Environmental Degradate*^a^					
	*3-PBA*	*4-F-3-PBA*	*cis-DBCA*	*cis-DCCA*	*trans-DCCA*	*MPA*
Bifenthrin						○^b^
Cyfluthrin		○		●	●	
λ-Cyhalothrin	●^c^					
Cypermethrin	●			●	●	
*cis*-Deltamethrin	●		○			
Esfenvalerate	●					
*cis*-Permethrin	●			●		
*trans*-Permethrin	●				●	

Abbreviations: 3-PBA (3-phenoxybenzoic acid); 4-F-3-PBA (4-fluoro-3-phenoxybenzoic acid); *cis*-DBCA (*cis*-3-(2,2-dibromovinyl)-2,2-dimethylcy-clopropane carboxylic acid); *cis*-DCCA (*cis*-3-(2,2-dichlorovinyl)-2,2-dimethylcyclopropane carboxylic acid); *trans*-DCCA (*trans*-3-(2,2-dichlorovinyl)-2,2-dimethylcyclo-propane carboxylic acid; MPA (2-methyl-3-phenylbenzoic acid).

a The environmental degradates are also commonly used as urinary biomarkers of exposure in humans.

b ’○’ equals specific metabolite of one pyrethroid insecticide.

c ’●’ equals nonspecific metabolite of one or more pyrethroid insecticides.

**Table 2.** Estimated limits of detection (LODs) and limits of quantitation (LOQs) for the target analytes in the duplicate-diet solid food samples.

*Target analyte*	*LOD (ng/g)*	*LOQ (ng/g)*
*Pyrethroid insecticide*		
Bifenthrin	0.05	0.10
Cyfluthrin	1.0	2.5
λ-Cyhalothrin	0.30	1.0
Cypermethrin	0.30	1.0
*cis*-Deltamethrin	0.05	0.25
Esfenvalerate	0.30	1.0
*cis*-Permethrin	0.10	0.25
*trans*-Permethrin	0.10	0.25
*Pyrethroid degradate*		
4-F-3-PBA	0.10	0.25
3-PBA	0.30	2.5
*cis*-DBCA	0.30	1.0
*cis*-DCCA	0.30	1.0
*trans*-DCCA	0.30	1.0
MPA	0.30	1.0

Abbreviations: 3-PBA (3-phenoxybenzoic acid); 4-F-3-PBA (4-fluoro-3-phenoxybenzoic acid); *cis*-DBCA (*cis*-3-(2,2-dibromovinyl)-2,2-dimethylcy-clopropane carboxylic acid); *cis*-DCCA (*cis*-3-(2,2-dichlorovinyl)-2,2-dimethylcyclopropane carboxylic acid); *trans*-DCCA (*trans*-3-(2,2-dichlorovinyl)-2,2-dimethylcyclo-propane carboxylic acid; MPA (2-methyl-3-phenylbenzoic acid).

**Table 3.** Levels of pyrethroid insecticides and their environmental degradates (ng/g) in the duplicate-diet solid food samples.

*Target analyte*	*N*^a,b^	*%*^c^	*90th*^d^	*95th*^d^	*Maximum*
*Pyrethroid insecticide*					
Bifenthrin	774	20	0.63	1.8	13.9
Cyfluthrin	781	1	—	—	103
λ-Cyhalothrin	781	2	—	—	27.7
Cypermethrin	781	7	—	1.6	154
*cis*-Deltamethrin	778	17	0.82	1.9	16.3
Esfenvalerate	780	2	—	—	358
*cis*-Permethrin	780	19	0.84	5.2	111
*trans*-Permethrin	774	21	0.90	5.4	172
*Pyrethroid degradate*					
4-F-3-PBA	781	<1	—	—	0.4
3-PBA	780	<1	—	—	2.3
*cis*-DBCA	779	<1	—	—	1.0
*cis*-DCCA	782	1	—	—	8.3
*trans*-DCCA	782	2	—	—	38.3
MPA	780	0	—	—	—

Abbreviations: 3-PBA (3-phenoxybenzoic acid); 4-F-3-PBA (4-fluoro-3-phenoxybenzoic acid); *cis*-DBCA (*cis*-3-(2,2-dibromovinyl)-2,2-dimethylcyclopropane carboxylic acid); *cis*-DCCA (*cis*-3-(2,2-dichlorovinyl)-2,2-dimethylcyclopropane carboxylic acid); *trans*-DCCA (*trans*-3-(2,2-dichlorovinyl)-2,2-dimethylcyclo-propane carboxylic acid; MPA (2-methyl-3-phenylbenzoic acid).

a Number of samples (*n* = 782).

b Missing data due to laboratory error.

c Percentage of analytes at or above the limit of detection by analyte.

d Percentile.

**Table 4.** Levels of the four most frequently detected pyrethroids in the duplicate-diet solid food samples.^a,b^

*Target analyte*	N^c^	%^d^	Mean±SD^e^	*Min.*	*50th*	*75th*^f^	*90th*^f^	*95th*^f^	*Max*.
Bifenthrin	156	100	1.8 ± 2.8	0.06	0.63	1.7	4.7	8.2	13.9
*cis*-Deltamethrin	129	100	1.9 ± 2.4	0.12	1.0	2.4	4.1	6.2	16.3
*cis*-Permethrin	145	100	9.1 ± 20.0	0.10	1.1	5.6	28.7	52.5	111
*trans*-Permethrin	164	100	8.9 ± 22.2	0.10	0.80	4.9	25.8	48.6	172

a Includes pyrethroids that were detected in ⩾ 17% of the food samples (Table 3). For each analyte, only values at or above the LOD are presented in this table.

b Units = ng/g.

c Number of samples.

d Percentage of samples.

e Arithmetric mean and SD.

f Percentile.

**Table 5.** The estimated adults’ maximum dietary exposures and intake doses to the target pyrethroid insecticides.^a^

*Pyrethroid*	*Maximum dietary exposure (ng/day)*	*Maximum dietary dose (ng/kg/day)*	*Oral RfD*^b^ *(ng/kg/day)*
Bifenthrin	6206	63.1	15,000
Cyfluthrin	31,153^c^	265	—^d^
λ-Cyhalothrin	7807	69.7	5000
Cypermethrin	46,207^c^	393	10,000
cis-Deltamethrin	205	2.93	—
Esfenvalerate	102,071	1784	—
Permethrin^e^	121,000	2115	50,000

a Estimates using 24-h duplicate-diet solid food samples (excludes beverages).

b Oral reference dose (RfD), US EPA’s Integrated Risk Information System (IRIS), http://www.epa.gov/iris/.

c Missing food mass data from same study participant due to laboratory error; used average food mass data of all study adults by time period.

d Not available in IRIS.

e Combined *cis*- and *trans*-isomers.

## References

[1] Elbert A, Haas M, Springer B, Thielert W, Nauen R. Applied aspects of neonicotinoid uses in crop protection. Pest Manag Sci 2008; 64: 1099–1105. 18561166 10.1002/ps.1616

[2] Saillenfait AM, Ndiaye D, Sabate JP. Pyrethroids: exposure and health effects – an update. Int J Hyg Envir Heal 2015; 218: 281–292. 10.1016/j.ijheh.2015.01.002 25648288

[3] USEPA (United States Environmental Protection Agency). Pyrethrins/ Pyrethroids Cumulative Risk Assessment. Available at: http://epa.gov/pesticides/cumulative/common mechgroups/htm#pyrethrins, 2011 Accessed on 29 July 2015.

[4] Riederer AM, Bartell SM, Barr DB, Ryan PB. Diet and nondiet predictors of urinary 3-phenoxybenzoic acid in NHANES 1999–2002. Environ Health Perspect 2008; 116: 1015–1022. 18709153 10.1289/ehp.11082 PMC2516573

[5] Barr DB, Olsson AO, Wong LY, Udunka SO, Baker SE, Whitehead RD Urinary concentrations of metabolites of pyrethroid insecticides in the general US population: National Health and Nutrition Examination Survey 1999–2002. Environ Health Perspect 2010; 118: 742–748. 20129874 10.1289/ehp.0901275 PMC2898848

[6] Morgan MK, Sheldon LS, Croghan CW, Jones PA, Chuang JC, Wilson NK. An observational study of 127 preschool children at their homes and daycare centers in Ohio: Environmental pathways to *cis*- and *trans*-permethrin exposure. Environ Res 2007; 104: 266–274. 17258193 10.1016/j.envres.2006.11.011

[7] Li W, Morgan MK, Graham S, Starr JM. Measurement of Pyrethroids and Their Environmental Degradates in Fresh Fruits and Vegetables using a Modification of the Quick Easy Cheap Effective Rugged Safe (QuEChERS) Method. Talanta 2016; 151: 42–50. 26946008 10.1016/j.talanta.2016.01.009

[8] Chuang JC, Wilson NK. Multiresidue analysis of organophosphate and pyrethroid pesticides in duplicate-diet solid food by pressurized liquid extraction. J Environ Sci Health B 2011; 46: 41–40. 20972922 10.1080/03601234.2010.515505

[9] Lu C, Schenck FJ, Pearson MA, Wong JW. Assessing children’s dietary pesticide exposure: Direct measurement of pesticide residues in 24-hr duplicate food samples. Environ Health Perspect 2010; 118: 1625–1630. 20639183 10.1289/ehp.1002044 PMC2974704

[10] Riederer AM, Hunter RE, Hayden SW, Ryan PB. Pyrethroid and organophosphorus pesticides in composite diet samples from Atlanta, USA adults. Environ Sci Technol 2010; 44: 483–490. 19994894 10.1021/es902479h

[11] Chen L, Zhao T, Pan C, Ross JH, Krieger RL. Preformed biomarkers including dialkylphosphate (DAPs) in produce may confound biomonitoring in pesticide exposure and risk assessment. J Agric Food Chem 2012; 60: 9342–9351. 22906185 10.1021/jf303116p

[12] Melnyk LJ, McCombs M, Brown GG, Raymer JJ, Nishioka MG, Buehler S Community duplicate diet methodology: A new tool for estimating dietary exposures to pesticides. J Environ Monit 2012; 14: 85–93. 22048778 10.1039/c1em10611b

[13] USDA (United States Department of Agriculture). Pesticide Data Program. Available at: http://www.ams.usda.gov/datasets/pdp. 2014 Accessed on 13 January 2016.

[14] USFDA (United States Food and Drug Administration). Total Diet Study. 2004–2005 Available at: http://www.fda.gov/Food/FoodScienceResearch/TotalDietStudy/ default.htm. Accessed on 13 January 2016.

[15] Leng G, Kuhn KH, Idel H. Biological monitoring of pyrethroids in blood and pyrethroid metabolites in urine; Applications and limitations. Sci Total Environ 1997; 199: 173–181. 9200861 10.1016/s0048-9697(97)05493-4

[16] Ratelle M, Cote J, Bouchard M. Toxicokinetics of permethrin biomarkers of exposure in orally exposed volunteers. Toxicol Lett 2015; 232: 369–375. 25498136 10.1016/j.toxlet.2014.12.003

[17] Eadsforth CV, Bragt PC, Van Sittert NJ. Human dose-excretion studies with pyrethroid insecticides cypermethrin and alphacypermethrin: Relevance for biological monitoring. Xenobiotica 1988; 18: 603–613. 3400277 10.3109/00498258809041697

[18] CDC (Centers for Disease Control and Prevention). Fourth national report on human exposure to environmental chemicals. 2009 Available at: http://www.cdc.gov/exposurereport/. Accessed on 11 August 2015.

[19] McKelvey W, Jacobson JB, Kass D, Barr DB, Davis M, Calafat AM Population-based biomonitoring of exposure to organophosphate and pyrethroid pesticides in New York City. Environ Health Perspect 2013; 121: 1349–1356. 24076605 10.1289/ehp.1206015 PMC3855501

[20] Trunnelle KJ, Bennett DH, Tulve NS, Clifton MS, Davis MD, Calafat AM Urinary Pyrethroid and Chlorpyrifos Metabolite Concentrations in Northern California Families and Their Relationship to Indoor Residential Insecticide Levels, Part of the Study of Use of Products and Exposure Related Behavior (SUPERB). Environ Sci Technol 2014; 48: 1931–1939. 24422434 10.1021/es403661a

[21] Morgan MK, Sobus JR, Boyd-Barr D, Croghan CW, Chen F, Walker R Temporal variability of pyrethroid metabolite levels in bedtime, morning, and 24-hr urine samples for 50 adults in North Carolina. Environ Res 2016; 144: 81–91. 26584066 10.1016/j.envres.2015.11.003

[22] Anastassiades M, Lehotay SJ, Stajnbaher D, Schenck FJ. Fast and easy multiresidue method employing acetonitrile extraction partitioning and dispersive solid phase extraction for determination of pesticide residues in produce. J AOAC Int 2003; 86: 412–431. 12723926

[23] Lehotay SJ, Mastovska K, Yun SJ. Evaluation of two fast and easy methods for pesticide residue analysis in fatty food matrixes. J AOAC Int 2005; 88: 630–638. 15859091

[24] Rosenblum L, Hieber T, Morgan J. Determination of pesticides in composite dietary samples by gas chromatography/mass spectrometry in the selected ion monitoring mode by using a temperature-programmable large volume injector with preseparation column. J AOAC Int 2001; 84: 891–900. 11417652

[25] Verbovsek T A comparison of parameters below the limit of detection in geochemical analyses by substitution methods. Materials Geoenviron 2011; 58: 393–404.

[26] IRIS (Integrated Risk Information System). 2015 Available at: http://www.epa.gov/iris/. Accessed on 21 August 2015.

[27] Schettgen T, Heudorf U, Drexler H, Angerer J. Pyrethroid exposure of the general population – is this due to diet? Toxicol Lett 2002; 134: 141–145. 12191872 10.1016/s0378-4274(02)00183-2

[28] Melnyk LJ, Xue J, Brown GG, McCombs M, Nishioka M, Michael LC. Dietary intakes of pesticides based on community duplicate diet samples. Sci Total Environ 2014; 468–469: 785–790. 24070872 10.1016/j.scitotenv.2013.08.101

[29] Morgan MK, Sheldon LS, Croghan CW, Chuang JC, Lordo R, Wilson NK A Pilot Study of Children’s Total Exposure to Persistent Pesticides and Other Persistent Organic Pollutants (CTEPP). United States Environmental Protection Agency: Washington, DC, USA, 2004 EPA/600/R-041/193.

[30] Matuszewski BK, Constanzer ML, Chavez-Eng CM. Strategies for the assessment of matrix effect in quantitative bioanalytical methods based on HPLC-MS/MS. Anal Chem 2003; 75: 3019–3030. 12964746 10.1021/ac020361s

