# Supplementary material for: Pyrethroid insecticides and their environmental degradates in repeated duplicate-diet solid food samples of 50 adults
Source: J Expo Sci Environ Epidemiol. Author manuscript; Available in PMC 2018 Aug 9. (PMC6084439; doi:10.1038/jes.2016.69)
Supplement: s1 [file NIHMS983415-supplement-s1.doc]

**Supplemental Information**

Analytical method performance was demonstrated through studies of recovery, inter-day precision, matrix effect, and detection limits (Table 2). For the recovery study, 20 aliquots of control matrix were spiked at 10 ng/g and processed in a single batch in the same manner as study samples. Average percent recoveries and standard deviations (SD) are given in Supplemental Table 4. To assess batch recovery performance, acceptance limits were established for each analyte based on a range that corresponded to the average recovery ± 3 SD with a modest expansion to account for preparation of the complex matrix by different personnel over time. Inter-day precision was determined by replicate analysis over 3 days of 5 samples spiked with all analytes at 10 ng/g. The relative standard deviation was less than 5% for all compounds with the exception of cyfluthrin (10.6%) and *λ-*cyhalothrin (6.46%). A matrix effects study was performed in the manner of Matuszewski *et al.* ^30^ The matrix effect value for all analytes was less than 100% and indicates ion suppression. The matrix effect values suggest the greatest ion suppression for DCCA (42.0%) and *cis*-DBCA (42.6%), and the least for *λ-*cyhalothrin (92.6%). Recovery efficiency ranged from 91.5% for deltamethrin to 105.3% for 3-PBA. Results for process efficiency ranged from 41.7% for DBCA to 92.2% for *λ-*cyhalothrin.

Overall, results for the laboratory control samples (LCS) were within empirically-determined acceptance limits and indicate that the analytical method generally was performing as expected for all analytical batches (Supplemental Table 4). Recovery results for matrix spike samples showed more variability compared to the control matrix results, as expected due to the high variability in composition of the field sample matrices. However, the mean percent recoveries of the field spike samples exhibited deviations from the acceptance limits for *λ-*cyhalothrin, *cis*-permethrin, *trans*-permethrin, 3-PBA, and *cis*-DBCA. Also, the mean percent recovery of *cis*-deltamethrin in the field spikes was at the low end of the acceptable recovery range. The observed decrease in *λ-*cyhalothrin*, cis-*permethrin, *trans*-permethrin, and *cis*-deltamethrin levels and the increase in 3-PBA and *cis*-DBCA levels in these field spike samples suggests that some degradation of these parent pyrethroids likely occurred during the collection phase and storage of the study samples. Mass balance calculations, however, indicate that the observed increases in 3-PBA and *cis*-DBCA levels in these samples were greater than contributions that could solely occur by degradation of the spiked parent compounds since the spike levels of the parent compounds (0.9, 4.5, and 8.9 ng/g) were five times lower than the spike levels of the degradation products (4.5, 22.5, and 45 ng/g) in the same sample. Therefore, the spiked levels of *cis*-permethrin, *trans*-permethrin and *cis*-deltamethrin would only account for about 10% of the total levels of these degradates measured in the field spike samples. Thus, the total increase in 3-PBA and *cis*-DBCA in these samples is likely due to additional unknown factors (i.e., interferences/matrix effects, presence of other pyrethroids that can breakdown to form 3-PBA, or chemicals of the same mass that fragment similarly). This information suggests the “true” concentrations of *λ-*cyhalothrin, *cis*-permethrin, *trans*-permethrin, and *cis*-deltamethrin in the participants’ food samples is likely higher (i.e., 2-fold) than what we reported in our current study. Conversely, the high bias observed for the degradation products, 3-PBA and *cis*-DBCA, suggest that the “true” concentrations in the study samples is likely lower than reported here. Note that the possibility and extent of bias to analyte concentrations in the study samples can only be estimated since the sample matrices differed from our control matrix. In addition, since previous diet studies^10,12^ did not collect field spike samples, it is possible that they may have underestimated the levels of pyrethroid residues in their adult food samples. This information emphasizes the importance of collecting both field and laboratory QC samples in human exposure measurements studies.

**Supplemental Table 1**. Summary of AB Sciex 4000 Qtrap parameters

| Parameter | Setting |
| --- | --- |
| Curtain Gas | 20 |
| Collision Gas | Low |
| Ion Spray Voltage (Pyrethroids, Degradates), V | 5500, -4500 |
| Source Temperature, °C | 450 |
| Ion Source Gas 1 | 45 |
| Ion Source Gas 2 | 45 |
| Interface Heater | On |
| Run Time, min. | 10 |
| Column Temperature, °C | 60 |
| Flow Rate (Pyrethroids, Degradates), mL/min | 0.400, 0.300 |

**Supplemental Table 2**. Mobile phase gradients

| Pyrethroids | | Pyrethroid Degradates | |
| --- | --- | --- | --- |
| Time  (min.) | Mobile Phase B  (%) | Time (min.) | Mobile Phase B (%) |
| 0 | 75 | 0 | 2 |
| 3.0 | 75 | 2.0 | 2 |
| 6.0 | 98 | 3.0 | 98 |
| 6.1 | 75 | 7.0 | 98 |
| --- | --- | 7.1 | 2 |

**^a^** The mobile phases for the parent pyrethroids were (A) 25:75 5 mM ammonium acetate:methanol in 0.1% formic acid and (B) 5 mM ammonium acetate in methanol with 0.1% formic acid. For the pyrethroid degradates, the mobile phases were the same as mentioned above, except formic acid was not included.

**Supplemental Table 3**. Summary of multiple reaction monitoring mode settings

| Analyte Transition | Precursor Ion, *m/z* | Fragment Ion, *m/z* | DP, V | EP*,* V | CE, V | CXP, V |
| --- | --- | --- | --- | --- | --- | --- |
| *cis-*DBCA Q1^a^ | 295.01 | 78.90 | -40 | -10.0 | -14 | -1 |
| *cis-*DBCA Q2^b^ | 295.01 | 80.80 | -45 | -10.0 | -16 | -1 |
| DCCA Q1^a^ | 206.94 | 35.10 | -60 | -10.0 | -34 | -3 |
| DCCA Q2^b^ | 208.94 | 37.10 | -55 | -10.0 | -34 | -3 |
| *trans*-DCCA IS Q1^a^ | 210.00 | 35.10 | -60 | -10.0 | -38 | -3 |
| 4-F-3-PBA Q1^a^ | 230.98 | 187.00 | -45 | -10.0 | -20 | -11 |
| 4-F-3-PBA Q2^b^ | 230.98 | 93.00 | -45 | -10.0 | -40 | -7 |
| 4-F-3-PBA IS Q1^a^ | 236.95 | 99.00 | -60 | -10.0 | -36 | -15 |
| MPA Q1^a^ | 211.03 | 167.00 | -60 | -10.0 | -18 | -1 |
| MPA Q2^b^ | 211.03 | 129.00 | -60 | -10.0 | -18 | -7 |
| 3-PBA Q1^a^ | 213.02 | 93.00 | -60 | -10.0 | -28 | -13 |
| 3-PBA Q2^b^ | 213.02 | 65.00 | -60 | -9.9 | -74 | -9 |
| 3-PBA IS Q1^a^ | 219.08 | 98.80 | -85 | -10.0 | -30 | -15 |
| Bifenthrin Q1^a^ | 439.895 | 181.10 | 61 | 10.0 | 17 | 12 |
| Bifenthrin Q2^b^ | 442.08 | 181.10 | 76 | 10.0 | 17 | 12 |
| Cyfluthrin Q1^a^ | 453.05 | 193.10 | 61 | 10.0 | 23 | 14 |
| Cyfluthrin Q2^b^ | 451.07 | 191.10 | 61 | 10.0 | 23 | 34 |
| Cyfluthrin IS Q1^a^ | 457.07 | 190.90 | 61 | 10.0 | 21 | 12 |
| λ-Cyhalothrin Q1^a^ | 467.13 | 225.00 | 71 | 10.0 | 25 | 14 |
| λ-Cyhalothrin Q2^b^ | 469.12 | 227.20 | 66 | 10.0 | 23 | 14 |
| Cypermethrin Q1^a^ | 433.10 | 191.00 | 56 | 10.0 | 21 | 12 |
| Cypermethrin Q2^b^ | 433.10 | 416.10 | 56 | 10.0 | 13 | 22 |
| Cypermethrin IS Q1^a^ | 439.05 | 191.00 | 56 | 9.9 | 21 | 12 |
| Deltamethrin Q1^a^ | 522.95 | 280.80 | 61 | 10.0 | 23 | 20 |
| Deltamethrin Q2^b^ | 520.94 | 278.99 | 61 | 10.0 | 23 | 18 |
| Esfenvalerate Q1^a^ | 437.10 | 167.00 | 61 | 10.0 | 23 | 10 |
| Esfenvalerate Q2^b^ | 439.10 | 169.00 | 61 | 10.0 | 23 | 18 |
| Permethrin Q1^a^ | 408.10 | 183.00 | 46 | 10.0 | 53 | 12 |
| Permethrin Q2^b^ | 410.10 | 183.00 | 46 | 10.0 | 53 | 12 |
| Permethrin IS Q1^a^ | 414.04 | 189.10 | 56 | 10.0 | 29 | 12 |

^a^ Transition used for quantitation.

^b^ Transition used for confirmation.

**Supplemental Table 4**. Summary of laboratory quality control and field spike recovery data

|  | Bifenthrin | Cyfluthrin | *λ-*Cyhalothrin | Cypermethrin | *cis-*Deltamethrin | Esfenvalerate | *cis-*Permethrin | *trans-*Permethrin | 3-PBA | 4F-3PBA | *cis-*DBCA | MPA | *trans-*DCCA |
| --- | --- | --- | --- | --- | --- | --- | --- | --- | --- | --- | --- | --- | --- |
| % Recovery Acceptance Range^a^ | 80-125 | 60-140 | 60-120 | 70-120 | 75-120 | 70-120 | 80-120 | 80-135 | 80-120 | 80-120 | 65-125 | 80-120 | 80-120 |
| Empirical % Recovery Range^b^ | 93.6±4.2 | 94.0±8.2 | 90.4±4.8 | 101.1±4.6 | 101.7±5.9 | 92.9±7.4 | 87.4±3.7 | 96.3±4.6 | 95.1±3.4 | 95.5±3.0 | 95.5±3.0 | 101.4±3.2 | 105.8±5.9 |
| Mean % Recovery, All Batch LCS^c^ | 104.2±13.4 | 97.9±12.3 | 94.9±16.3 | 93.6±7.5 | 94.6±11.9 | 89.6±10.4 | 97.8±10.2 | 94.4±12.0 | 97.4±9.8 | 99.2±6.8 | 95.7±13.3 | 98.0±9.2 | 100.8±9.6 |
| Mean % Recovery, All Batch MSS^d^ | 89.2±23.2 | 101.4±18.4 | 95.5±16.9 | 95.8±12.0 | 93.4±14.1 | 94.0±30.7 | 94.4±17.2 | 96.5±16.4 | 99.3±7.8 | 101.8±8.8 | 91.1±20.9 | 102.4±24.0 | 102.7±8.4 |
| Mean % Recovery, Medium and High Level Field Spikes^e^ | NS^f^ | 73.3±39.8 | 58.5±24.8^g^ | 100.0±34.0 | 81.5±30.1 | 99.4±34.5 | 47.4±17.9 ^g^ | 53.8±22.1^g^ | 162.8±61.4^g^ | 105.0±40.8 | 184.7±77.6^g^ | NS^f^ | 107.5±37.7 |

^a^ Range used for determining acceptable recovery which was based on results of our recovery study.

^b^Average recovery ± 3 standard deviations obtained our recovery study performed using the control matrix spiked with all target analytes at 10 ng/g.

^c^ Laboratory control sample (LCS) consisted of the control food mixture spiked with target analytes at 10 ng/g.

^d^ Matrix spike sample (MSS) consisted of a study food sample spiked with target analytes at 10 ng/g. Analyte recovery was calculated using the observed amount in the MSS minus the average amount observed in the corresponding study sample and LD.

^e^ Two field spike samples were excluded from this dataset due to laboratory preparation error.

^f^ Not spiked; we obtained the bifenthrin and MPA standards after preparation of the field spike samples.

^g^ Percent recovery is outside method acceptance range.
